# Effects of fearful face presentation time and observer’s eye movement on the gaze cue effect

**DOI:** 10.1186/s40101-023-00325-4

**Published:** 2023-05-29

**Authors:** Chuntai Yu, Keita Ishibashi, Koichi Iwanaga

**Affiliations:** 1 Graduate School of Science and Engineering, Chiba University, 1-33 Yayoi-Cho, Inage-Ku, Chiba, 263-8522 Japan; 2 Design Research Institute, Chiba University, 1-33 Yayoi-Cho, Inage-Ku, Chiba, 263-8522 Japan

**Keywords:** Gaze cueing effect, Fear facial expression, Spatial attention, Electrooculography, Amygdala

## Abstract

**Background:**

There are many conflicting findings on the gaze cueing effect (GCE) of emotional facial expressions. This study aimed to investigate whether an averted gaze, accompanied by a fearful expression of different durations, could enhance attentional orientation, as measured by a participant’s eye movements.

**Methods:**

Twelve participants (3 females) completed the gaze cue task, reacting to a target location after observing changes in the gaze and expression of a face illustrated on a computer screen. Meanwhile, participants’ eye movements were monitored by electrooculography. The GCE was calculated by reaction time as an indicator of attention shift.

**Results:**

The analysis of the overall data did not find a significant effect of fearful facial expressions on the GCE. However, analysis of trial data that excluded a participant’s eye movement data showed that brief (0, 100 ms) presentation of the fearful facial expression enhanced the GCE compared to that during a neutral facial expression, although when the presentation time of the fearful expression was increased to 200 or 400 ms, the GCE of the fearful expression was at the same level as when model showed a neutral expression.

**Conclusions:**

The results suggest that the attention-enhancing effect of gaze cues induced by rapidly presented fearful expressions occurs only when the effect of eye movement trials is excluded. This effect may be mediated by reflexively neural circuits in the amygdala that process threatening stimuli. However, as the expression duration increased, the fearful expression’s attention-enhancing effect decreased. We suggest that future studies on the emotion modulation of GCE should consider the negative effects of participants’ saccades and blinks on the experimental results.

## Background

Humans are regarded as social animals, and facial information is a social cue necessary for smooth interindividual communication. Facial information such as facial expressions and gaze directions can effectively convey the expresser’s mental state, such as interest, intention, and emotion, to other individuals [1]. Humans have the highest proportion of exposed white sclera among primates, making their eyes structurally suitable for eye gaze communication [2, 3]. As a result, humans are more sensitive to another person’s gaze direction, which conveys information about important events in the environment. By reading and interpreting gaze direction, the observer detects and adjusts spatial attention accordingly [4].

Numerous studies [5–10] have verified a reflexive reaction, called the gaze cueing effect (GCE), that orients one’s attention to the direction of another person’s gaze. The GCE is generally considered to promote the sharing of attention between two people on the same object (joint attention), which is a crucial ability to promote interindividual social interaction.

Friesen et al. [6] modified the Posner task into the gaze-cueing task, which examined whether participants shifted their attention to gaze the direction. In this task, a facial stimulus (gaze cue) that looks to the left or right was first presented as a cue in the center of a screen, and a target was presented on either the same side (congruent) or opposite side (incongruent) of the gaze cue. Participants responded to the target position by pressing a button to indicate which side of the screen the target was on, and the reaction time and correct answer rate were recorded. Participants generally detected targets faster when the target was congruent rather than incongruent with the gaze cue. The difference in reaction time between incongruent and congruent trials reflected the magnitude of gaze-evoked attentional orientation as the GCE.

Understanding emotional facial expressions is another important skill in guiding social interactions [11]. Humans appear to share neural circuits for processing gaze and facial expressions, and studies have shown that the brain regions for processing gaze direction and affective perception information are highly correlated and interact during activation [12, 13]. Fearful and angry facial expressions are considered threat-related, and previous studies have reported involuntary processing of threatening information [14, 15]. The combination of fearful expression and gaze may signal a direction of threat in the environment, resulting in humans shifting their concentration of attention more rapidly to adapt to a dangerous situation.

However, Hietanen and Leppänen [16] conducted a series of experiments and reported that facial expressions (neutral, happy, angry, fearful) did not affect the GCE. Mathews et al. [17] reported that fearful expressions had a significantly larger GCE than neutral expressions only in the individuals with high anxiety level. They interpreted this as anxious people being more sensitive to information about potential threats.

In subsequent studies [18–20], a non-anxious group and a general group also reported that facial expression affected the GCE, contradicting previous findings. Graham et al. [18] conducted experiments using dynamic stimuli presentation methods ranging from neutral to emotional gazes (e.g., fear, disgust, pleasure). They found that the reaction time was shorter for emotional gazes than for neutral ones and that the magnitude of the GCE was greater in response to emotional expressions than to neutral ones. Lassalle and Itier [19, 20] considered the effect of the presentation order of gaze and facial stimuli. In a stimulus presentation sequence in which the expression changed from neutral to fearful after a gaze change, the fearful expression enhanced the GCE. This sequence of stimulus cues is considered to have high ecological validity, meaning it is close to what might happen in a real environment. Therefore, by using this stimulus sequence, the enhanced attentional shifts in fearful expressions can be correctly induced.

One factor that has contributed to the conflicting results of previous studies is thought to be fearful expression presentation time. In studies of spatial cues, an important factor called stimulus onset asynchrony (SOA) which meaning the length of time between a cue stimulus and a target presentation. It has been reported that SOA affects the GCE. A short SOA (150, 300 ms) produces a robust GCE, while the GCE losses in long SOA show that an automatic shift of attention characterizes the GCE [6]. McCrackin and Itier [21] reported that the effects of fearful facial expressions could occur at a 200-ms SOA when a stimulus presentation sequence is used in which the facial expression changes from neutral to fear after a gaze change; this result supported the theory of reflexive information processing by the fearful expression. However, Graham et al. [18] argued that 300 ms was not enough time for the integration of gaze and facial expression, as no effect of fearful expression was seen on the GCE in SOA of less than 300 ms.

Gaze direction and fearful expression appear to be integrated in the early period of visual information processing. The presentation time may affect the threat message formed by the combination of fearful expression and averted gaze. A short presentation time may be perceived as to indicate an obvious threat, while a long one may be judged as ambiguous regarding the presence of a threat. Therefore, the presentation time of fearful expression may affect the intensity of the perceived threat and thus affect the spatial attention shift of gaze direction.

In typical gaze cue tasks, SOA refers to the length of time from gaze presentation to target presentation. In a dynamic gaze cueing task testing the effect of the fearful expression, it is necessary to set the SOA as the total presentation time of the gaze cue and the fearful expression. However, due to the different experimental task designs in previous studies, their results cannot be directly compared. Therefore, it is necessary to investigate the effect of facial expression presentation time on the effect of fearful expression rather than simply to consider SOA as a factor.

Moreover, the inconsistency of findings among the previous studies may be related to differences among the participants’ eye movements from experiment to experiment [22, 23]. Bannerman et al. [24] reported that fearful expressions evoked faster and more saccades in participants than neutral expressions. Therefore, the emotional content of a presented face may induce spontaneous eye movements in participants. Visual exploration in primates depends on saccadic eye movements that cause alternations of neural suppression and enhancement [25]. Saccades will inevitably divert spatial attention, affecting the target's reaction time and thereby affecting the GCE. Moreover, unlike the manual response, the saccade response will induce the earlier inhibition of attention shift in the cue direction (inhibition of return) to develop earlier [26]. To the best of our knowledge, however, only one study has investigated the effect of saccade on emotion modulation in GCE. McCrackin et al. [27] investigated the effect of saccades generated during a gaze cueing task. In that study, happy expression elicited a greater GCE than neutral tongue trials when eye movements were removed but not when they were included. For the GCE enhancement effect of the fearful expression, there was no difference in the results from before and after the eye movement trial was removed. In the present study, while excluding saccades, we also focused on the effect of eye blinking on the experimental results because blinks may interrupt visual information input. In simple terms, eye blinking may affect the perception of the presentation time of the fearful expression, resulting in a negative influence on experimental data. McCrackin et al. [27] reported on the proportion of trials that included saccades but not on the proportion that included blinking. To better discuss the effect of the fearful expression on participants’ spontaneous eye movements, it is necessary to report the number of blink trials in the experiment and to discuss the effect of blinking on the experimental results.

Electrooculography (EOG) is a physiological measurement of eye movements that records changes in the cornea‒retina potential [28]. Vertical eye movements (blinks) can be measured by placing electrodes on the lids for VEOG (vertical EOG). Meanwhile, horizontal eye movements (saccades) can be measured by placing electrodes on the external canthi for HEOG (horizontal EOG). Since the EOG technique can simultaneously measure horizontal eye movement saccades and vertical eye movement blinks with a high temporal resolution and can be synchronized with the event signal of the experimental task, we believe that EOG is a suitable measurement of eye movements for investigating the influence of eye movement occurring during the execution of the gaze cueing task in this study design.

In summary, we conducted an experiment to explore the effects of fearful expression presentation time and of a participant’s eye movements on the GCE of fearful and neutral expressions to expand our understanding of the attention-shifting behavior evoked by fearful expressions.

## Methods

### Participants

The sample size was estimated based on prior studies on the effects of facial expression (*η*_*p*_^*2*^ = 0.22) and of SOA on reaction time (*η*_*p*_^*2*^ = 0.86) [21]. A statistical power analysis using G*Power [29] indicated an optimal sample size of *N* = 8 if the effect size *η*_*p*_^*2*^ = 0.2 with *α* = 0.05 and power = 0.95.

Twelve university students (3 female) with normal vision (including corrected-to-normal vision) participated in the experiment. The age of the participants was 25.0 ± 1.7 years (M ± S.D.), and all were right-handed. All participants gave written informed consent before participating in the experiment. The experimental implementation sequence of this study was approved by the ethics committee of The Institute of Engineering and Center for Frontier Medical Engineering of the Graduate School at Chiba University (Acceptance Number: R2-11).

### Stimuli

Two male and two female head models of neutral and fear facial expressions with a straight gaze were generated by 3D facial model generation software (FaceGen Modeller 3.5, Singular Inversions). The fearful expression was created using the Facial Action Coding System (FACS) built in FaceGen Modeller, which specifies a set of Nos. 1, 2, 4, 5, 7, 20, and 25 action units (AUs). All these AUs were considered characteristics of fearful expression [30, 31]. By using 3D model rendering software (Blender, Blender Foundation), facial images were created with the gaze of each model oriented to the left or right 20° from the straight gaze. Each stimulus was trimmed to a size of 12.6° × 20°, showing the region from the top of the head to the upper neck.

The stimuli were presented by a program created by PsychoPy3.0 on a 24.5-inch LCD monitor (ROG SWIFT PG258Q, 1920 × 1080 pixels, 240 Hz, ASUS) connected to a Windows 10 computer against a dark gray (64, 64, 64 RGB) background. Participants observed the stimulus at 50 cm from the LCD and responded using a keyboard. A chinrest ensured a fixed distance of 50 cm from the computer screen and minimized participants’ head movements.

### Procedure

The experiment included three factors: facial expression, congruency, and duration of facial expression presentation, including two levels of expression conditions (fear and neutral expressions) and three levels of gaze–target congruency (congruent, incongruent, and straight gaze). In addition, four durations of facial expression presentation (0, 100, 200, and 400 ms) were set. Because the straight gaze condition was set as a pseudo-condition, it was excluded from the analysis. Participants completed a practice block and five experimental blocks. Participants completed 24 trials in the practice block (one trial for each experimental condition). Each experimental block contained 8 repetitions for the left and right gaze directions and 4 repetitions for the straight gaze direction. As a result, there were 128 trials in each of the 16 conditions (2 expressions × 2 gaze – target congruency × 4 durations of facial expression presentation) and 32 trials in the straight gaze condition (2 expressions × 4 durations of facial expression presentation) for each experimental block, lasting about 8 min. Participants were allowed to take proper rest periods between blocks. Thus, participants completed the experiment in approximately 60 min.

**Fig. 1:**
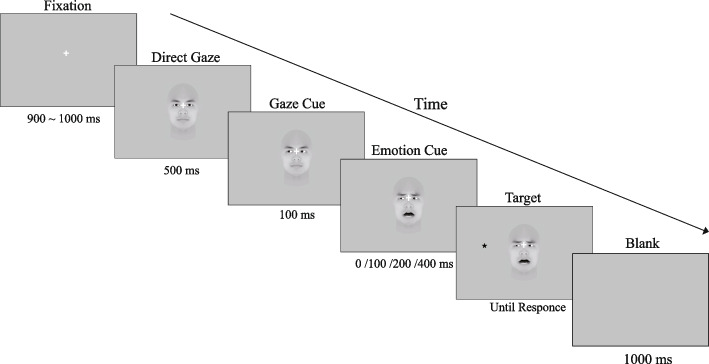
Sequence of experimental stimuli. This diagram shows only one example of facial and target conditions (fearful-congruent condition)

Each trial began with a central fixation point prompted at a random time of 900 ~ 1000 ms, in which a neutral expression facial image with a straight gaze was presented for 500 ms. After each neutral presentation, a gaze cue consisting of an image of a face with a left, straight, or right gaze was presented within 100 ms to present a perception of a gaze shift. Second, as emotional cues, the facial expression either remained neutral or changed to fear and was presented for 0, 100, 200, or 400 ms. Finally, the asterisk target (2.5° × 2.5°) appeared on the left or right side of the facial stimulus. The target would randomly appear either left or right of the face. Participants were instructed to respond to the location of the target (left or right) as quickly and accurately as possible by pressing the “left arrow” or “right arrow” key with their right index finger and middle finger. Participants were also asked to keep their eyes fixed on a certain fixation cross during the completion of the experiment. Each participant’s reaction time (RT) was recorded (see Fig. 1).

The gaze direction was either congruent with the target location (looking toward the location where the target would later appear) or incongruent with it (looking in the opposite direction). Left and right gaze trials were averaged within the two gaze-congruency conditions.

The period from the start of the fixation point to the end of the participant’s response was considered as one trial, and the subsequent trial was started after a blank picture was presented for 1000 ms. Participants were informed that they must maintain their focus on the fixation point until a blank frame appeared.

### EOG measurements and data classification criteria

EOG was recorded to measure the participant's eye movements at a sampling rate of 1000 Hz and visualized in real-time using biological signal analysis software (AcqKnowledge 4.1, Biopac Systems) Two electrodes were placed on the left and right temples (horizontal eye movement detection), two were placed above and below the right eye (vertical eye movement detection), and a reference electrode was placed on the forehead.

Before the experiment began, each participant was instructed to maintain a fixed gaze at the fixation point, and baseline EOG was recorded in both the horizontal and vertical directions for 5 s. During the duration of the experimental stimulus between the Fixation frame and the Target frame (see Fig. 1), if the maximum amplitude in the horizontal or vertical EOG was greater than the maximum amplitude in the baseline, it was considered a saccade (horizontal) or a blink (vertical) trial, whereas if the maximum amplitude in the horizontal or vertical EOG was not greater than that in the baseline, it was considered a non-eye-movement trial. We synchronized these classification markers to the reaction time data before data analysis.

### Statistical analysis

Statistical analyses were conducted using SPSS Statistics (Chicago, IL, USA). The correct RTs were analyzed separately for all trials, including both eye movement trials and non-eye movement trials, by using a repeated measures analysis of variance (ANOVA) with within-subject factors of facial expression (neutral and fear), congruency (congruent and incongruent), and facial expression presentation time (0, 100, 200, and 400 ms).

We also performed an ANOVA on the GCE, which was calculated as the difference between incongruent and congruent RTs (RT*incongruent* − RT*congruent*) using facial expression and facial expression presentation time as within-subject factors.

Statistical significance was accepted at the 5% level (*p* < 0.05). The Greenhouse‒Geisser correction was applied where sphericity was violated.

## Results

Table 1 shows the exclusion rates resulting from each participant’s eye movement, error response, and deviation of RT. Trials in which an incorrect response was made (mean = 0.7%, SD = 0.6%) and those in which the participant’s mean RT was above or below 3 SDs (mean = 3.1%, SD = 1.7%) were removed before the mean for each condition was calculated. Because we aimed to investigate the influence of a participants’ eye movement on the experimental results, we compared the analyses of data including and excluding eye movement trials.

**Table 1:** Data exclusion rates resulting from eye movement, error response, and deviation of RT

Data exclusion rates (%)				
***Participant***	Blink eye movement	Total eye movement	Error response	Deviation of RT above or below 3 SDs
1	7.7	12.0	1.0	2.3
2	4.0	4.3	0.1	1.8
3	22.8	27.5	0.0	5.3
4	9.8	12.4	0.6	6.7
5	13.1	15.0	0.3	3.1
6	4.8	7.0	1.4	1.3
7	11.5	13.5	0.1	1.5
8	16.7	18.0	1.0	1.4
9	7.0	9.5	0.6	4.4
10	10.7	13.6	0.3	2.3
11	10.1	10.4	1.8	3.5
12	1.4	2.3	0.6	3.1
**Mean (SD)**	10.0 (5.8)	12.1 (6.6)	0.7 (0.6)	3.1 (1.7)

Values show the percentages of 800 trials (160 trials × 5 blocks) that were excluded

### Analysis of all trials including eye movement trials

A 2 (facial expression: “neutral”; “fear”) × 2 (congruency: congruent; incongruent) × 4 (facial expression presentation time: 0, 100, 200, and 400 ms) repeated measures ANOVA was conducted on the mean reaction times of total data including eye movements trials.

The results showed a main effect of congruency (*F* (1, 11) = 20.57, *p* < 0.001, *η*_*p*_^*2*^ = 0.65), as participants responded to gazed-at (congruent) targets were more quickly than to non-gazed-at (incongruent) targets. However, there was no main effect of facial expression (*F* (1, 11) = 1.52, *p* = 0.244, *η*_*p*_^*2*^ = 0.12) or of facial expression presentation time (*F* (3, 33) = 2.05, *p* = 0.126, *η*_*p*_^*2*^ = 0.15). No significant interaction was observed between facial expression and congruency (*F* (1, 11) = 0.01, *p* = 0.942, *η*_*p*_^*2*^ = 0.004), between congruency and facial expression presentation time (*F* (3, 33) = 0.6, *p* = 0.62, *η*_*p*_^*2*^ = 0.05), or between facial expression and facial expression presentation time (*F* (3, 33) = 1.21, *p* = 0.321, *η*_*p*_^*2*^ = 0.10).

**Fig. 2:**
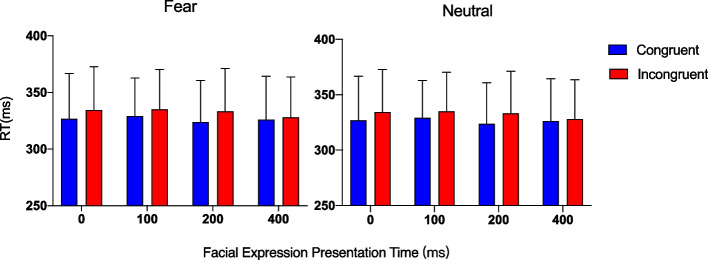
Mean reaction time (RT) for each facial expression presentation time and fear (left) and neutral (right) expressions for all trial data. Error bars indicate standard errors of the mean

Finally, the three-way interaction was not significant (*F* (3, 33) = 1.41, *p* = 0.256, *η*_*p*_^*2*^ = 0.11). As the main effect of and interaction with facial expression did not show statistical significance, the effect of the fearful expression on the GCE was not examined (see Fig. 2).

### Analysis of trials excluding eye movement trials

As described above, a three-way repeated measures ANOVA was conducted on mean reaction times, excluding the data of eye movement trials.

**Fig. 3:**
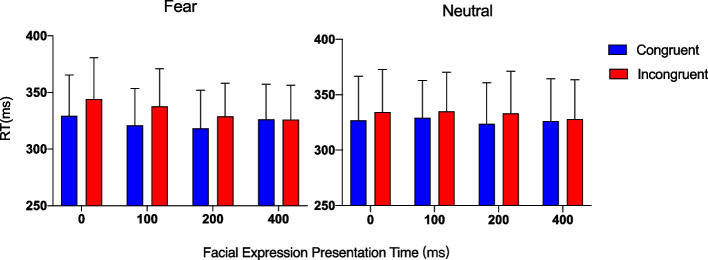
Mean reaction time (RT) for each facial expression presentation time and fear (left) and neutral (right) expressions when the eye movement trials data were excluded. Error bars indicate standard errors of the mean

Congruency had a significant main effect (*F* (1, 11) = 68.14, *p* < 0.001, *η*_*p*_^*2*^ = 0.86), the same as when the analysis of eye movements was included, but reflects that the effects were stronger when eye movement data were excluded. Facial expression presentation time (*F* (1.55, 17.06) = 3.82, *p* = 0.051, *η*_*p*_^*2*^ = 0.26) had a significant main effect. Although the main effect of facial expressions (*F* (1, 11) = 0.13, *p* = 0.728, *η*_*p*_^*2*^ = 0.01)was not well established, significant interactions were observed between facial expression and congruency(*F* (1, 11) = 9.60, *p* = 0.01,* η*_*p*_^*2*^ = 0.46), congruency and facial expression presentation time(*F* (3, 33) = 14.48, *p* = 0.001,* η*_*p*_^*2*^ = 0.57), and facial expression and facial expression presentation time(*F* (3, 33) = 4.08, *p* = 0.014,* η*_*p*_^*2*^ = 0.27). Finally, the three-way interaction was significant (*F* (3, 33) = 3.05, *p* = 0.026,* η*_*p*_^*2*^ = 0.24). These interaction results showed that the effect of facial expression was modulated by facial expression presentation time and congruency (see Fig. 3).

Since a significant three-way interaction was found between facial expression, congruency, and facial expression presentation time, we conducted ANOVAs on the congruent and incongruent trials separately. Results showed that for RT in congruent trails, facial expressions had a certain trend toward a significant main effect (*F* (1, 11) = 3.50, *p* = 0.088, *η*_*p*_^*2*^ = 0.24). No significant main effect of facial expression presentation time was found (*F* (1.78, 19.62) = 2.26, *p* = 0.135, *η*_*p*_^*2*^ = 0.17), but the interaction between facial expression and facial expression presentation time approached the borderline of significance (*F* (3, 33) = 2.64, *p* = 0.076, *η*_*p*_^*2*^ = 0.19). Next, we conducted a simple main effect analysis for each factor. The Bonferroni correction (two-tailed) was applied to tests of statistical significance of post-hoc comparison, and the significance level was set at 0.008. Simple main effect analysis of facial expression showed shorter RTs for fearful than for neutral trials when the fearful expression was prompted 100 ms or 200 ms before the target (100 ms condition: 321.1 ms vs 329.1 ms, *F* (1, 11) = 8.83, *p* = 0.013,* η*_*p*_^*2*^ = 0.44; 200 ms condition: 318.3 ms vs 323.7 ms, *F* (1, 11) = 5.80, *p* = 0.035, *η*_*p*_^*2*^ = 0.35). The simple main effect of facial expression presentation time was also significant in the fearful expression condition (*F* (3, 33) = 7.80, *p* = 0.002,* η*_*p*_^*2*^ = 0.41). A further multiple comparison reflected that the RT was longer for the 0 ms condition than for the 100 or 200 ms condition (*ps* < 0.005), and RT for the 200 ms condition was shorter for the 400 ms condition (*p* < 0.005). For RT in incongruent trials, there was a significant main effect of facial expression presentation time (*F* (1.64, 18) = 7.91, *p* = 0.005, *η*_*p*_^*2*^ = 0.42) and significant interaction between facial expression and facial expression presentation time (*F* (3, 33) = 5.50, *p* = 0.007, *η*_*p*_^*2*^ = 0.33), but the main effect of facial expression was not observed (*F* (1, 11) = 0.51, *p* = 0.487, *η*_*p*_^*2*^ = 0.04). Simple main effect analysis of facial expression showed longer RTs for fearful than for neutral trials only when the fearful expression was prompted simultaneously with the target (0 ms condition: 344.2 ms vs 334.5 ms, *F* (1, 11) = 9.54, *p* = 0.01, *η*_*p*_^*2*^ = 0.46). The simple main effect of facial expression presentation time in the fearful expression condition was observed (*F* (3, 33) = 11.84, *p* = 0.001, *η*_*p*_^*2*^ = 0.52), reflecting that the RT was longer for the 0 ms and 100 ms conditions than for the 200 ms or 400 ms condition (*ps* < 0.005). There was no significant difference between the 0 ms and 100 ms conditions or between the 200 ms and 400 ms conditions.

As congruency interacted with every other factor, the magnitude of GCE was computed (RT*incongruent* − RT*congruent*) and analyzed by using a 2 (facial expression) × 4 (facial expression presentation time) repeated measures ANOVA. When the interaction is significant, the Bonferroni correction (two-tailed) was applied to tests of statistical significance in the post-hoc comparison for the simple main effect of facial expression presentation time, and the significance level was set at 0.008.

**Fig. 4:**
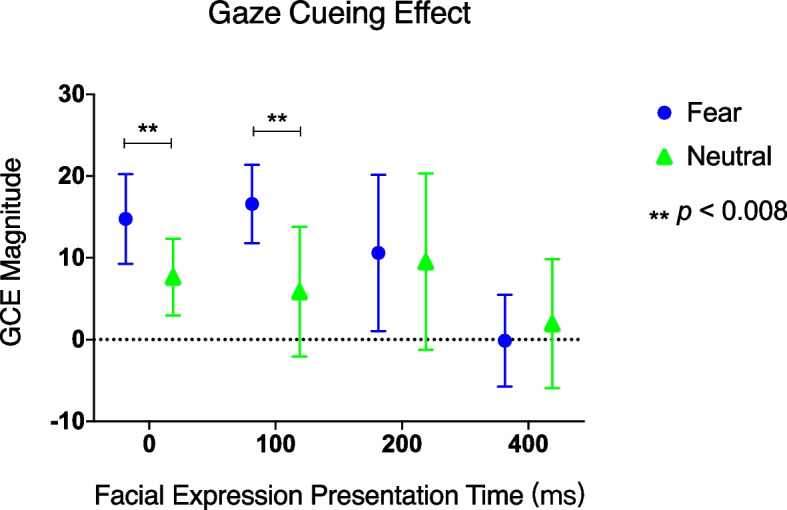
Mean gaze cueing effect (i.e., RT difference between congruent and incongruent trials) for each facial expression presentation time among neutral and fearful expressions when the eye movement trial data were excluded. Error bars indicate standard errors of the mean. Asterisks indicate *p* < 0.008 (the threshold after Bonferroni correction)

First, significant main effects of facial expression (*F* (1, 11) = 9.53, *p* = 0.01, *η*_*p*_^*2*^ = 0.46) and facial expression presentation time (*F* (1, 11) = 14.52, *p* < 0.001,* η*_*p*_^*2*^ = 0.57) were found. Moreover, their interaction was also significant (*F* (3, 33) = 3.49, *p* = 0.05, *η*_*p*_^*2*^ = 0.24). The simple main effect analysis of facial expression showed that when the fearful expression was prompted simultaneously with or 100 ms before the target, the fearful expression had a significantly greater effect than the neutral expression (0 ms condition: 14.8 ms vs 7.7 ms, *F* (1, 11) = 21.06, *p* < 0.001,* η*_*p*_^*2*^ = 0.66; 100 ms condition: 16.6 ms vs 5.9 ms, *F* (1, 11) = 42.19, *p* < 0.001,* η*_*p*_^*2*^ = 0.79). The simple main effect of facial expression presentation time was also significant in the fearful expression condition (*F* (3, 33) = 16.27, *p* < 0.001, *η*_*p*_^*2*^ = 0.59), reflecting that the GCE was larger for the 0, 100, and 200 ms conditions than for the 400 ms (*ps* < 0.008, see Fig. 4).

## Discussion

Humans can exploit social information such as gaze and facial expressions, enabling each other to communicate and transmit information more efficiently [32, 33]. Previous research has debated whether threatening information expressed by fearful facial expressions can enhance attention shift evoked by gazing at a target, but no consistent conclusion has been reached [16, 18, 27]. The present study set out to assess the importance of two factors, facial expression presentation time and participants’ spontaneous eye movements, that affect the enhancement of GCE by a fearful expression. The results demonstrated that this enhancement of GCE by a fearful expression was found only in the specific condition where the two main factors were combined (i.e., fearful expression under the short presentation time condition and excluding the results of the spontaneous eye movement trials). In two separate subsections in the “ Discussion” section, we discuss and summarize the effects of these two factors on the GCE enhancement of fearful expressions and their potential causation.

### Facial expression presentation time

Across the present experimental results, we demonstrated that fearful expressions enhance gaze-evoked spatial attention shifts compared to neutral expressions. This effect is moderated by the presentation time of fearful expressions. However, this attentional enhancement effect of fearful expressions was found only in the results after excluding the data from spontaneous eye movement trials.

When presentations were brief (i.e., 0 and 100 ms), the GCE of the fearful expression was greater than that of the neutral expression. The greater GCE can be achieved in two ways: by pointing more quickly to the target in congruent conditions, or more slowly to the target in incongruent conditions. The present study showed that, in congruent trials lasting 100 or 200 ms, participants responded faster to the target when it was preceded by a fearful expression compared to a neutral expression. In addition, the attentional enhancement effect was more pronounced for the 100 ms duration than for the 200 ms duration, suggesting that attention was more rapidly directed towards the gaze cue when a fearful expression was presented. This is consistent with previous findings that fearful expressions speed up the orientation of spatial attention compared to neutral expressions [21]. In incongruent trials, when the fearful face and the target were presented simultaneously (0 ms condition), the response to the target was slower for the fearful expression compared to the neutral expression. This was the underlying reason for the enhancement of the GCE by the fearful expression in the 0 ms condition. We consider that findings from previous studies [34, 35] may explain this result. Carlson and Mujica-Parodi [34] investigated the impact of fearful facial expressions on spatial attention during conscious and unconscious information processing using a dot-probe task. They found that, regardless of conscious awareness, the presentation of fearful facial threat cues resulted in both faster orientation of attention towards the cued location and slower disengagement of attention from it. Georgiou et al. [35] suggested that spatial attentional processes may also act to delay disengagement from fear-relevant stimuli, even though this tendency is stronger among people with high anxiety. The present study adds to previous research by demonstrating that simultaneously presenting a fearful expression and a target impairs participants' ability to disengage their attention from the gaze-cued side associated with the potential threat. Specifically, the findings suggest that this effect is more pronounced for fearful expressions compared to neutral faces. In summary, the results of the present study confirm that when the fearful expression and the target were presented simultaneously after the gaze cue, the enhancement effect of GCE may be due to the difficulty in disengagement of attention from the gaze direction. When the fearful expression lasting for 100 ms, the GCE enhancement may be due to the faster shift of attention to the eye direction. However, the RT results for congruent trials in this study approached, but did not reach, statistical significance, thus limiting the interpretation of the results in the present experiment. This lack of statistical significance could be due to the relatively small sample size compared to previous studies, even though a statistical power analysis was performed before the experiment. Moreover, the present study did not measure or control for individual anxiety levels, which may also be a limitation in interpreting the results of this study. Therefore, future studies should aim to verify the results of this study by increasing the sample size and controlling individual anxiety levels.

A fearful face with averted gaze is often considered an indicator of threatening information. For example, a person looking to the right and expressing fear suggests there may be a danger in that direction. The ability to integrate and understand these cues, interpreted as an essential survival mechanism, is thought to reside in the amygdala, which is part of the limbic system and located deep inside the brain’s medial temporal lobe.

The amygdala integrates sensory information and outputs it to the hypothalamus. In these neural networks, the amygdala plays a vital role in judging the value of information to survival [36]. Thus, the amygdala processes and remembers emotional responses (especially fear and anger) and may have pathways for processing threat-related signals [37]. In an fMRI study, Adams et al. [12] showed that expressions related to threat information, such as fearful expression, could induce a stronger reaction in the amygdala than the neutral expression and could promote information processing of fearful expression. In addition, previous studies have shown that fear emotion is detected through the limbic system; especially, threat information related to survival will be quickly and automatically processed to get ready for the following action [38, 39].

Kawashima et al. [40] demonstrated that gaze information is also processed in the amygdala. The detection of gaze direction activates the left amygdala, which helps read social signals. Presenting fearful expressions and averting gaze in a short period of time may induce rough and rapid information processing in the amygdala, integrating fearful expressions and gaze direction to judge whether there is a threat to survival. As a result, fearful expression will cause spatial attention to be distributed more quickly to the gaze direction.

In amygdaloid processing of information about a threatening stimulus, two different neural circuits may be at work: one for reflexive responses and the other for reflective. By operating in parallel, these circuits can not only detect the existence of a threat rapidly and roughly but can also cancel the threat after confirming the safety of carrying out the most efficient threat response [41]. By adjusting the presentation duration of fearful expressions, Adams et al. [42] found that a short presentation induced a reflexive response in the amygdala to explicit threat information, while a long presentation induced a reflective response in the amygdala to an ambiguous threat. Our results support the hypothesis that when explicit threat information (such as the fearful expression with a specific direction of gaze in this study) is prompted for a short time (100 ms), the threat detection circuit of the amygdala is reflexively activated. The signal is quickly transmitted to the spatial attention system to guide attention rapidly. This supports the view that the fearful expression induced an automatic attention shift in previous studies. Most noteworthy is that, based on the results of previous studies, the present study is the first to confirm that the GCE was enhanced when the fearful face and the target were presented simultaneously, although no expression effect on congruent RT was found. Future research may continue to explore the GCE enhancement effect of fearful expression when the presentation time is within 0–100 ms.

However, when the presentation time is longer, but the target still does not appear, it may be temporarily considered that there is no threat (or ambiguous threat) in the environment, the top-down processing of the cortex affects spatial attention, and the GCE by fearful expression is not enhanced. Such results support the hypothesis of the processing of threatening stimuli: that when attention shifts to the gaze cue direction and no target is found, the spatial attention will deviate from the cue direction to respond to targets appearing in other directions in the environment. Such results could also reflect human environmental adaptability, as a long-term focus on a specific direction is detrimental to detecting environmental hazards [43].

In the present study, SOA duration was the time between the gaze cue and the target appearance. Hence, the SOA factors in this study were 100, 200, 300, and 500 ms for both fear and neutral expressions. However, the fearful expression occurred for 0, 100, 200, and 400 ms after the gaze shift (lasting 100 ms), while the neutral expression was maintained for 0, 100, 200, and 400 ms. Even though this is a classic experimental design for investigating emotion modulation in GCE, the lack of apparent facial motion in neutral expressions could potentially have affected the results; this may be a limiting aspect of this study. To eliminate this potential impact in future studies, we expect to create an experimental condition like the ‘neutral tongue’ in McCrackin et al.’s study [21, 27].

While the present study discussion involves a lot of speculation about brain circuits that process the gaze of fearful expressions, obviously, it is not enough to speculate on the neural basis behind the GCE enhancement by the fearful expression based only on the reaction time data. Therefore, we believe it may be possible to further confirm the conjecture by introducing neurophysiological methods in future studies.

### Spontaneous eye movements

The comparison between total data and eye movement removal data revealed that a participant’s eye movements during the experiment affected the detection of the GCE enhanced by fearful expression.

Similar to the results reported by McCrackin et al., [27] in our study the effect of fearful expression on the GCE can be clarified by excluding from analysis the data on eye movements recorded during trials. In contrast, when all of the data were analyzed, no effect of fearful expression was found. The results of the present study showed that the effect of fearful expressions could be detected with a small sample size (12 participants) if the data affected by eye movements were excluded, even though the effect of fearful expressions is generally considered to be small. As we mentioned in the Background section, Graham et al. [18] argued that a 300-ms cueing time was insufficient to integrate gaze and facial expressions, as they found no effect of fearful expressions on the GCE at shorter SOAs. However, the present study demonstrated that, when the cueing time of fearful expressions was less than 100 ms (i.e., when SOA was less than 200 ms in the present study), fearful expressions enhanced the GCE. When the cueing time of fearful expressions exceeded 100 ms (i.e., when SOA exceeded 200 ms in the present study), the enhancement effect of fearful expressions on the GCE disappeared, contradicting previous findings. Compared to the experimental approach of Graham et al. [18], the present study required participants to fixate their gaze during the experiment and eliminated experimental data that had been contaminated by eye movements. This elimination of contamination might have contributed to the detection of the enhanced GCE of fearful expression by brief presentation. Conversely, when data analyses that did not account for the effects of eye movements were performed, no effect of fear expressions on the GCE was observed.

There are generally considered to be two types of orientation of visual attention: one is covert visual attention orientation, which is achieved through internal neurophysiological systems without head or eye movements; the other is overt visual attention orientation through behavioral systems accompanied by head or eye movements [44, 45]. During saccadic eye movements, visual nerve activity is suppressed [25], and an earlier inhibition of return for spatial attention shift is developed [26]. Prior studies have revealed that both overt and covert attention can induce GCE [46], but studies on the enhancement of GCE by fearful expression have not reached consistent conclusions. In the present study, the participants were asked to keep their eyes fixed on a certain point during the completion of the experiment, and to focused mainly on the effect of covert attention. However, since it is difficult to accurately locate the spatial position of the participants’ eye movements during the horizontal eye movements measured in this study, we cannot discuss the effect of overt attention of GCE enhancement by fearful expression. Therefore, this study focused on the relationship between covert attention and GCE enhancement by the fearful expression through strict exclusion criteria for eye movement trials. The present results confirm that the enhancement of GCE by fearful expression may be partially achieved by covert attention, which precedes eye movements and can be deployed simultaneously in multiple places in the environment. This mechanism is also in line with the advantages of threatening stimuli described above and is very helpful for efficient visual information processing and for guiding future eye movements. Nevertheless, the present results do not enable us to discuss whether the GCE enhancement by fearful expression is dominated by covert attention or the result of the combination of covert and overt attention. This is a limitation of the present study. Future studies need to explore their effects separately through experimental design.

However, unlike the case in previous studies, most of the eye movements in this study were caused by eye blinks (about 82.6% in total eye movements), which have the potential to temporarily obscure visual information and interrupt the continuity of visual information input, thus affecting the perceived prompt time [47]. A blank temporarily interrupts the flow of visual information between the world and the retina. In that instant, visual stimuli from the outside world disappear for 150–400 ms [48]. As mentioned above, the GCE enhancement by fearful expression is sensitive to the expression’s presentation time. Therefore, the blinks that occurred during the stimulus sequence may affect the perception of the presentation time of the fearful expression and thus affect the GCE enhancement by the fearful expression. As shown in the removal rate of trials including the eye movements of each participant (Table 1), we found some individual differences in the control of eye movements during the experiment. This phenomenon may be explained by differences in the cognitive resources that participants devote to the experiment. Maffei et al. [49] reported that participants individually had different blink rates depending on the difficulty of task execution, which showed that participants induced low probability of blinking in tasks with high difficulty and a high probability of blinking in tasks with low difficulty. It was also reported that, regardless of the task's difficulty, eye fatigue increased gradually during a 4 min task, and the blink rate increased accordingly. Considering the influence of such visual fatigue on eye movement, in future research tasks should be designed for completion within 4 min in one block.

## Conclusions

This study found that when a fearful gaze appeared at the same time as a target or 100 ms before the target, participants focused more intensely on the gaze direction than when the gaze was neutral. However, as the expression duration increased, the attention-enhancing effect of the fearful expression attenuated. Such results were seen only in analyses of data without eye movement. The GCE enhancement effect of the fearful expression is dominated by a faster response to the target in a short period of time. Hence, the present results supported that threat information may automatically orient attention at an unconscious level. We suggest that in future studies that explore the influence of facial expressions on the GCE, a subject’s eye movements during the experiment should be considered by improving the experimental design or eye movement measurement.

## Data Availability

The datasets used and/or analyzed during the current study are available from the corresponding author on reasonable request.

## Abbreviations

EOG: Electrooculography
fMRI: Functional magnetic resonance imaging
GCE: Gaze cueing effect
RT: Reaction time
SD: Standard deviation
SOA: Stimulus onset asynchrony
